# The influence of challenge research stressors on research creativity among Chinese doctoral students: a mediated moderation model

**DOI:** 10.3389/fpsyg.2023.1290342

**Published:** 2023-11-01

**Authors:** Chunlei Liu, Min Wu, Xiaoqing Gao

**Affiliations:** ^1^School of Educational Science, Hunan Normal University, Changsha, China; ^2^School of International Education, Guangxi University of Science and Technology, Liuzhou, China

**Keywords:** challenge research stressors, research creativity, achievement motivation, supervisor developmental feedback, doctoral students

## Abstract

The research creativity of doctoral students is not solely fueled by their intrinsic motivation, but also thrives in an environment that offers challenging research opportunities, substantial support, and feedback from significant others. Based on the job demands-resources model, this study aims to explore the impact of challenge research stressors on the research creativity of Chinese doctoral students. A mediated moderation model was constructed to examine the mediating effect of achievement motivation and the moderating effect of supervisor developmental feedback on the relationship between challenge research stressors and research creativity. A total of 538 valid questionnaires were collected from doctoral students using convenience sampling and snowball sampling. The questionnaires included the Challenge Research Stressors Scale, the Research Creativity Scale, the Achievement Motivation Scale, and the Supervisor Developmental Feedback Scale. Regression analyses, bootstrap testing, and simple slope analyses were used to estimate the various relationships. The findings indicated that challenge research stressors had a positive effect on doctoral students’ research creativity. Supervisor developmental feedback positively moderated the impact of challenge research stressors on the achievement motivation and research creativity of doctoral students. Achievement motivation partially mediated the influence of challenge research stressors on doctoral students’ research creativity, and further fully mediated the interaction effect of challenge research stressors and supervisor developmental feedback on doctoral students’ research creativity. These findings contribute not only to our understanding of the mechanisms and boundary conditions through which challenge research stressors impact the research creativity of doctoral students, but also provide valuable insights into how to stimulate and maintain their research creativity.

## Introduction

As the “tip of the pyramid” of the national education system, doctoral education bears the vital mission of generating original scientific research outcomes, nurturing high-level innovative talents, and contributing to the nation’s scientific and technological self-reliance and innovation. Its significance and role in national revitalization through science, education, talent empowerment, and innovation-driven development have become increasingly prominent (Li and Xue, 2021). Among these innovative talents, doctoral students serve as a vital reserve and driving force in building an innovative nation. Thus, enhancing the research creativity of doctoral students has emerged as a top-priority training objective across numerous nations and academic institutions (Whitelock et al., 2008; Brodin, 2018; Breslin, 2019). Nevertheless, in recent years, due to the continuous expansion of doctoral enrolment in China, the quality of doctoral graduate training has seen a significant decline (Zhang et al., 2023). More specifically, the lack of research creativity among doctoral students has not been able to keep up with the demands of social development (Su et al., 2021). Yuan and Yan (2009) discovered that the main issue affecting the quality of China’s postgraduate education is the lack of innovative ability, particularly in terms of originality. In their three separate surveys evaluating the quality of postgraduate education in China, nearly one-third of doctoral students rated their creativity as “average” or “poor.” Given this challenging scenario, it is of substantial practical significance to explore the factors that influence the research creativity of doctoral students and the underlying mechanisms. This exploration aims to improve the quality of doctoral student development and ignite their passion for and commitment to innovation.

Previous research has primarily focused on examining the factors that impact the research creativity of doctoral students from various perspectives, including supervisors, doctoral students themselves, and organizational climate. The supervisor level focuses on factors such as supervisor support (Fan et al., 2019; Zeng and Zhang, 2022; Zhang et al., 2023), supervisor mentoring style (Su et al., 2021; Zeng and Zhang, 2022; Zhang et al., 2023), supervisor competence (Zheng et al., 2022), and supervisor-student relationship (Ma et al., 2019; Zhao et al., 2021). The individual perspective focuses on the impact of doctoral students’ research experience (Brodin, 2014; Yin et al., 2018), personality traits (Wu et al., 2018; Su and Zhang, 2020), role identity (Yin et al., 2016; Frick and Brodin, 2020), research self-efficacy (Pan and Gu, 2022; Xie et al., 2023), and academic emotions (Yin et al., 2016) on their research creativity. The organizational climate perspective focuses on factors such as the climate for error management (Yin et al., 2018), the academic interaction atmosphere (Wu et al., 2019), and the organizational innovative environment (Liu et al., 2023) on doctoral students’ research creativity. Although the above studies have comprehensively discussed the factors influencing the research creativity of doctoral students, they have neglected to include the characteristics of the scientific research work that doctoral students are facing.

In recent years, with the continuous expansion of doctoral enrolment, doctoral students are facing increased academic and employment competition, where “no publication means elimination” (Horta and Li, 2022). Challenge research stressors, such as time constraints, substantial responsibilities, heavy research workloads, and research complexity, prevail in the doctoral research environment (Mccauley and Hinojosa, 2020; Acharya et al., 2023; Bran et al., 2023). Especially with the increasing expectations of the country on the quality of doctoral education, various institutions have raised the bar for the research and innovative abilities of doctoral students. Most universities in China require doctoral students to publish high-quality academic papers in order to be eligible for graduation, which adds to the already significant research pressure faced by doctoral students. As an inevitable and significant contextual factor in doctoral research, research stressors have a profound impact on the psychological cognition and behavioral outcomes of doctoral students during their research activities (El-Ghoroury et al., 2012). For example, existing studies have begun to examine the impact of research stressors on doctoral students’ anxiety (Yao and Ma, 2021), knowledge-sharing behaviors (Li et al., 2018), academic misconduct behaviors (Zhang et al., 2013), and research performance (Gu and Chang, 2021). However, there remains a gap in understanding the mechanisms through which research stressors affect the research creativity of doctoral students. Since previous studies on the impact of work stressors on individual creativity have mainly focused on the field of business management, little is known about how research stressors in academic organizations affect the creativity of graduate students. Therefore, it is of great significance to explore the relationship between research stressors and the research creativity of doctoral students in academic organizations.

Based on existing literature, scholars generally classify stressors into challenge stressors and hindrance stressors based on the two-dimensional stressors framework proposed by Cavanaugh et al. (2000). It has been found that hindrance stressors cannot be overcome by individuals in the short term and positively predict cognitive resource depletion, emotional exhaustion (Sawhney and Michel, 2022), and reduced self-efficacy (Sun et al., 2019; Yang and Li, 2021), thus exerting a negative effect on individual creativity (Horan et al., 2020; LePine, 2022). However, regarding the impact of challenge stressors on individuals’ creativity, scholars have reached inconsistent research conclusions, finding both positive (Sacramento et al., 2013), negative (Binnewies and Wörnlein, 2011; Wijaya et al., 2022), and nonlinear relationships (Baer and Oldham, 2006) between them. In light of this, drawing upon insights from the management field, this study focuses on investigating the influence of challenge research stressors in the field of doctoral education on the research creativity of doctoral students and the specific mechanisms involved.

Early studies have indicated that challenge stressors not only directly impacted individual creativity, but also exerted their influence through various mediating mechanisms (Lepine et al., 2005). For example, existing studies have predominantly explored and tested the mediating effects of variables such as self-efficacy (Sun et al., 2019), challenge appraisal (Ohly and Fritz, 2010), organizational commitment (Montani et al., 2017), regulatory focus (Wu et al., 2021) and emotions (Rodell and Judge, 2009) on the relationship of challenge stressors and individual creativity. However, according to Amabile’s componential theory of creativity (Amabile and Pratt, 2016), individual creativity is primarily derived from intrinsic motivation. This includes a strong interest and engagement in work, as well as a sense of curiosity, pleasure, or challenge associated with the work (Amabile et al., 1994). In essence, the willingness of individuals to engage in creative work and sustain this state hinges predominantly on their intrinsic motivation. Intrinsic motivation stands as a pivotal personal characteristic for enhancing creativity (Schoen, 2015). Based on this, the study posits that the research creativity of doctoral students primarily stems from their intrinsic identification with, strong interest in, and passion for academic research. That is, the intrinsic motivation of doctoral students plays a critical role in the formation and development of their research creativity. Drawing from achievement motivation (Weiner, 1985), achievement motivation represents a type of intrinsic motivation that drives individuals to pursue desired goals and overcome challenges in their pursuit of achievement. It includes the desire for success (a feeling of competence and accomplishment upon achieving goals) and the avoidance of failure (a strong aversion to taking risks and experiencing failure) (Collins et al., 2004). The inclination to pursue success exerts a positive influence on problem-solving and creativity (Story et al., 2009) and ranks among the most relevant individual factors associated with creativity (Schoen, 2015). Moreover, Yao and Ma (2021) have highlighted that challenge research stressors, such as time constraints, high research innovation requirements, and substantial research workloads, can positively predict postgraduates’ achievement motivation. Hence, based on this rationale, we propose that achievement motivation may act as a mediator in the relationship between challenge research stressors and doctoral students’ research creativity.

Furthermore, the Job Demands-Resources (JD-R) model theory suggests that achieving a balance between job demands and job resources is essential for generating positive outcomes (Bakker et al., 2007). Specifically, individuals who have access to sufficient job resources tend to exhibit higher levels of work motivation, enthusiasm, engagement, and a willingness to explore new things when confronted with high levels of job demands (Lesener et al., 2020). This, in turn, increases work creativity. Conversely, individuals lacking external resources, such as social support and feedback, are more likely to experience emotional exhaustion and anxiety when facing increased job demands (Han et al., 2020), which will hinder them from generating creative ideas and engaging in innovative behavior (Bakker and Demerouti, 2007). Based on the above, this study proposes that supervisors, as the primary individuals responsible for doctoral training, play a crucial role in providing support and feedback to help doctoral students effectively cope with demanding research requirements. Early studies have also shown that supervisors’ support and guidance are significant factors in predicting the innovative ability of doctoral students in research (Zheng et al., 2022). Therefore, it is worthwhile to explore whether supervisors’ feedback, especially the developmental feedback that focuses on students’ future growth and offers valuable insights (Zhou, 2003), can help doctoral students deal with challenge research stressors and enhance their intrinsic motivation for research and then improve their research creativity. Specifically, can supervisor developmental feedback positively moderate the impact of challenge research stressors on doctoral students’ achievement motivation and research creativity?

To summarize, this study aims to develop a mediated moderation model that examines the role of supervisor developmental feedback as a moderator and achievement motivation as a mediator in the relationship between challenge research stressors and research creativity among doctoral students. The purpose of this study is to provide strategies and recommendations to doctoral students, supervisors and doctoral training institutions on how to effectively manage challenge research stressors while also fostering and maintaining the research creativity among doctoral students.

## Literature review and hypotheses development

### Challenge research stressors and doctoral students’ research creativity

Creativity is a complex and diverse concept (Kandler et al., 2016), encompassing over 60 definitions within psychology alone (Elshout, 1990). Among the numerous definitions, Amabile’s proposal is widely accepted in academia, defining creativity as the ability to generate novel and appropriate ideas, products, processes, services, or methods (Amabile et al., 1996; Amabile, 1997). This definition has received extensive citation in research on individual-level creativity (Tierney et al., 1999; Zhou and George, 2001; Farmer et al., 2003). Existing studies have shown that individual creativity in the workplace is influenced by personal characteristics, organizational contextual factors, and their interaction (Oldham and Cummings, 1996; Shalley et al., 2004). When environmental characteristics align harmoniously with personal attributes, it tends to stimulate heightened levels of individual creativity (Woodman et al., 1993; Oldham and Cummings, 1996). In this study, creativity is referred to as research creativity, which pertains to the ability of doctoral students to systematically apply theoretical knowledge, creatively solve problems, and generate new insights. It also emphasizes the novelty and practicality of research questions, methodologies, processes, and perspectives (Yin et al., 2016).

The cognitive appraisal theory of stress suggests that stress is a psychological and physiological response produced by individuals after perceiving specific environmental demands and making either challenge or threat appraisals (Lazarus, 1993). Building upon this theory, Cavanaugh proposed a two-dimensional stressors framework comprising challenge stressors and hindrance stressors (Cavanaugh et al. 2000). Challenge stressors, such as time constraints, workload, job responsibilities, and task complexity, have the potential to foster personal growth and future development. In the specific research context of doctoral students, their research stress mainly comes from challenge research stressors, such as high research assessment requirements, substantial workload, tight deadlines, and the complexities of innovative research tasks. These stressors have the potential to reward doctoral students by improving their research abilities and fostering future academic growth. Once these stressors are overcome, doctoral students will experience positive rewards and a sense of accomplishment in relation to their research outcomes. This, in turn, will ignite their enthusiasm and intrinsic motivation for academic research. Based on this, we define challenge research stressors as research demands that fall within the acceptable range for doctoral students but require significant efforts to meet. These demands serve as motivators, prompting doctoral students to increase their commitment to research, and to propose and solve problems creatively.

Lepine et al. (2005) point out that coping with challenge stressors can elicit negative emotions such as tension and anxiety. However, once successfully managed, these stressors can provide opportunities for personal growth, learning, and future benefits. Meta-analyses investigating the challenge-hindrance stressors framework also consistently demonstrate that challenge stressors significantly and positively predict positive attitudes and behaviors in employees, including job satisfaction, organizational commitment, and job performance (Podsakoff et al., 2007; Horan et al., 2020; LePine, 2022). Based on this, it is hypothesized in this study that challenge research stressors positively predict the research creativity of doctoral students. Firstly, challenge research stressors contain attainable research objects and high research expectations (Podsakoff et al., 2007), which serve as motivating factors that inspire doctoral students to actively participate in research activities. This active engagement fosters a sense of achievement and efficacy (Sacramento et al., 2013), thereby fulfilling their psychological needs for competence. Self-determination theory suggests that satisfying individuals’ basic psychological need for competence in a certain activity will enhance their intrinsic motivation to engage in that activity (Ryan and Deci, 2000). Therefore, the sense of research efficacy derived from overcoming challenge research stressors will enhance doctoral students’ academic enthusiasm to actively seek resources, identify connections between concepts, and generate novel ideas (Farmer et al., 2003), ultimately promoting their research creativity. Secondly, although meeting the requirement of challenge research stressors demands significant effort from doctoral students, these challenge research stressors contain potential rewards for enhancing their research abilities and future academic growth. Therefore, doctoral students can gain a sense of control over challenging research requirements, which then help meet their psychological needs for autonomy. A meta-analysis by Hammond et al. (2011) indicates that autonomy is an important factor influencing individual innovative behavior. Individuals with higher autonomy have stronger adaptability and initiative in creative activities (De Spiegelaere et al., 2016). This is particular evident when research autonomy is granted to doctoral students, enabling them to independently select research topics and explore different research methods. As a result, they are able to generate more creative ideas. Based on these analyses, this study proposes the following hypothesis:

*H1:* Challenge research stressors have a positive impact on the research creativity of doctoral students.

### The mediating role of achievement motivation

Achievement motivation, as an important intrinsic trait, drives individuals to tackle meaningful, valuable, and challenging tasks with interest, enjoyment, and high self-confidence, cultivating inner motivation for successful outcomes (Wigfield and Eccles, 2000). It acts as an internal force propelling individuals toward success, reflecting their belief in self-development (Schoen, 2015). Individuals with high achievement motivation demonstrate greater proactivity and resilience when facing obstacles. They willingly take on more demanding tasks, invest substantial effort in achieving their goals (Wigfield and Eccles, 2000), and are more inclined to embrace risks and propose innovative solutions when confronted with problems. Moreover, they tend to positively evaluate associated risks, fostering a creative approach to their work (Dweck, 1986). Additionally, individuals with a strong need for achievement do not adhere to traditional solutions but instead focus on situations where existing solutions are inadequate. They consider these situations as opportunities to learn new knowledge and engage in challenging work (Shalley, 1995). Therefore, they are often seen as creative (Fodor and Carver, 2000), in alignment with the core tenet of the componential theory of creativity: creativity stems from intrinsic motivation rooted in their interest, enjoyment, or sense of challenge, with intrinsic motivation serving as the primary predictor of individual creativity (Amabile et al., 1996). Hence, the presence of achievement motivation significantly contributes to fostering individual creativity (Amabile, 1988; Shalley and Oldham, 1997). Building upon these insights, this study proposes that doctoral students’ achievement motivation can also positively predict their research creativity.

On the other hand, achievement motivation is also influenced by contextual factors. According to cognitive appraisal theory, organizational contextual factors can be classified as either informative or controlling. Informative contextual factors have a positive impact on intrinsic motivation, whereas controlling contextual factors have negative effects (Deci et al., 1989). Although challenge research stressors sometimes bring about negative emotions such as tension and anxiety, these stressors have the potential to reward doctoral students by improving their research abilities and fostering future academic growth (Lepine et al., 2005). Therefore, challenge stressors are deemed informative, providing doctoral students with relevant information to enhance their research abilities, thereby boosting intrinsic motivation and research creativity (Crawford et al., 2010). For instance, Wallace et al. (2009) found that challenge work stressors can serve as catalysts for individuals’ motivation to successfully complete tasks. Similarly, challenge research stressors, as an informative situational factor, will help ignite enthusiasm and subjective initiative among doctoral students, ultimately enhancing their commitment to research and intense passion for research innovation. Overcoming these challenges subsequently promotes a sense of competence and satisfaction, which in turn enhances motivation to achieve desired outcomes. Lepine et al. (2004) further noted a similar influence in college students who faced challenge academic stressors, with these stressors positively impacting their motivation to learn. Therefore, our study proposes that challenge research stressors serve as positive predictors of the achievement motivation experienced by doctoral students.

Given the above analysis, which is based on the componential theory of creativity and the cognitive appraisal theory of stress, individuals’ perception of work characteristics can influence their intrinsic motivation for task performance, subsequently impacting their creativity (Shalley et al., 2004). Specifically, in this study, challenge research stressors continually stimulate the desire for exploration and achievement among doctoral students. This stimulation enhances their intrinsic motivation for achievement, which, in turn, improves their curiosity, cognitive flexibility, adventurous spirit, and perseverance (Utman, 1997). Consequently, this promotes the development of their research creativity. Thus, we propose the following hypothesis:

*H2:* Achievement motivation mediates the relationship between challenge research stressors and doctoral students’ research creativity.

### The moderating role of supervisor developmental feedback

Feedback, in organizational contexts, serves as both a motivator and a corrective tool (Amabile et al., 2004; Christensen-Salem et al., 2018). Different from traditional results-oriented feedback or control-oriented feedback, which focuses on assessing behaviors and performance, developmental feedback provides valuable guidance for future learning, growth, and advancement (Zhou, 2003; Li et al., 2011). Therefore, developmental feedback is forward-looking and aims to facilitate improvement. This study defines developmental feedback from academic supervisors as the act of providing doctoral students with useful and valuable feedback information to enhance their learning, development, and improvement based on the idea of developmental feedback in organizational behavior.

Supervisors are critically important in guiding and supporting doctoral students throughout their academic socialization process (Gill and Burnard, 2008). Their developmental feedback significantly impacts the research cognition and behavior of doctoral students (Shang et al., 2023). The Conservation of Resources (COR) theory suggests that individuals with more resources are less affected by resource loss and can acquire additional resources (Hobfoll, 1989), potentially leading to a spiral of resource gain (Hobfoll, 2011). When doctoral students encounter challenge research stressors, receiving developmental feedback from their supervisors can potentially equip them with additional resources and mitigate the associated risks (Bakker et al., 2007). Firstly, in challenging research situations, constructive advice from supervisors enhances the research efficacy and self-confidence of doctoral students (Shang et al., 2023). This positive reinforcement leads to a more optimistic outlook on research challenges, inspiring proactive responses and boosting intrinsic motivation for demanding research tasks. Ultimately, it enhances research creativity. Previous studies have shown that supervisor developmental feedback strongly correlates with doctoral students’ intrinsic motivation and research creativity (Su et al., 2022). Notably, supervisors’ feedback carries greater influence on students’ research cognition and behavior compared to other sources of feedback (Gemme and Gingras, 2012). Secondly, supervisor developmental feedback primarily focuses on learning and improvement, emphasizing the provision of guiding advice for students’ future growth and progress (George and Zhou, 2007). Through receiving developmental feedback, doctoral students are able to gain insight into their strengths and research challenges. They also have access to valuable resources and information that contribute to their own academic growth (Zhou, 2003), which, in turn, stimulates their intrinsic motivation to pursue research achievements (Dweck, 1986), leading them to actively seek challenges, persevere, and generate creative ideas. As a result, their research creativity is enhanced. Additionally, supervisor developmental feedback, as an informative feedback approach, is not outcome-oriented, which helps alleviate the research stress of doctoral students and stimulates their interest in research (Joo and Park, 2010). That is, developmental feedback fosters a relaxed research environment that encourages divergent thinking and ultimately cultivates higher research creativity (Runco and Acar, 2012). Moreover, supervisors’ timely developmental feedback conveys relevant information about doctoral students’ future research, reflecting supervisors’ expectations, encouragement, and support. According to the Pygmalion effect, individuals who have higher positive external expectations tend to have stronger intrinsic motivation for success (Tierney and Farmer, 2004), which enhances their creative problem-solving abilities. Based on the above analysis, we propose the following hypotheses:

*H3:* Supervisor developmental feedback positively moderates the relationship between challenge research stressors and doctoral students’ achievement motivation, such that the positive relationship is stronger when supervisor developmental feedback is higher.

*H4:* Supervisor developmental feedback positively moderates the relationship between challenge research stressors and doctoral students’ research creativity, such that the positive relationship is stronger when supervisor developmental feedback is higher.

Integrating H2–H4, the present study further proposes the mediated moderating effect of supervisor developmental feedback. In other words, the interaction between supervisor developmental feedback and challenge research stressors affects doctoral students’ research creativity through the mediation of achievement motivation. Specifically, the more developmental feedback supervisors provide, the more instrumental and emotional support doctoral students will receive, and the better they will be able to recognize the positive aspects of challenge research stressors. This will stimulate the intrinsic motivation of doctoral students to pursue success in scientific research, which in turn will enhance their creativity in scientific research to a greater extent. Conversely, doctoral students may perceive a lack of care and support when they receive limited developmental feedback from supervisors. This perception can hinder their ability to recognize the positive aspects of challenge research stressors, ultimately reducing their confidence and motivation to achieve research goals. As a result, the effectiveness of challenge research stressors in stimulating research creativity through enhanced achievement motivation is weakened. In view of this, the following hypotheses are proposed:

*H5:* The positive moderating effect of supervisor developmental feedback on the relationship between challenge research stressors and doctoral students’ research creativity is mediated by achievement motivation.

**Figure 1:**
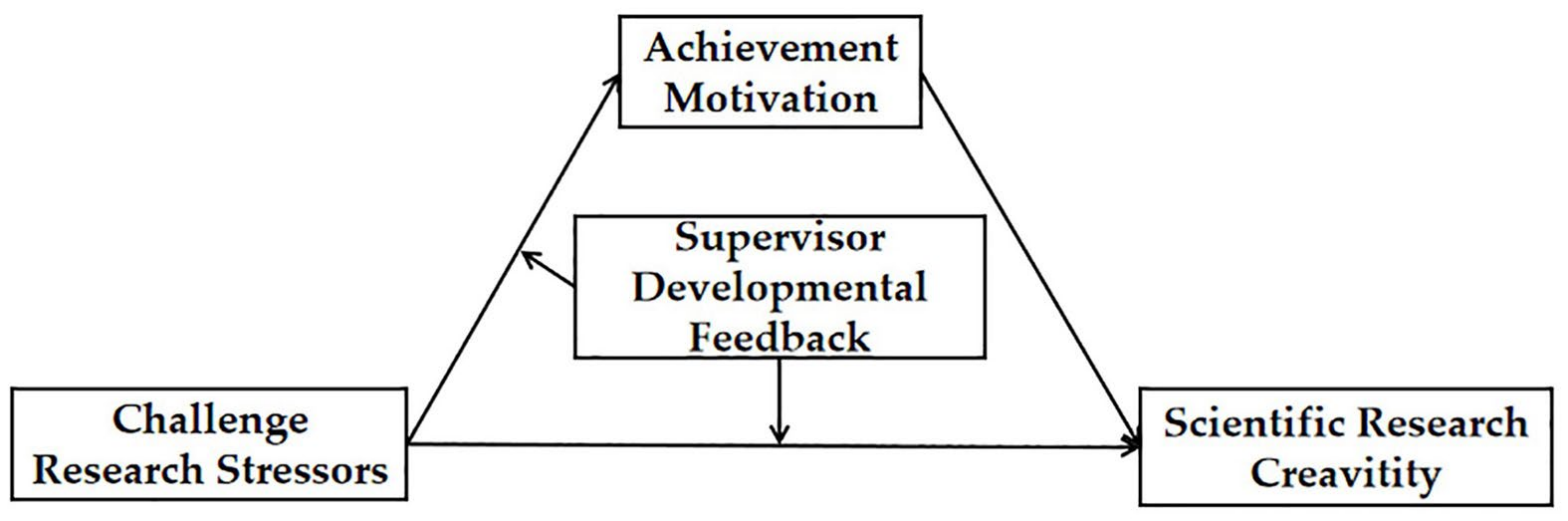
Proposed model.

In summary, this study developed a mediated moderation model (Figure 1) with challenge research stressors as the independent variable, research creativity as the dependent variable, achievement motivation as the mediator, and supervisor developmental feedback as the moderator.

## Materials and methods

### Participants and procedure

In this study, we imported the prepared questionnaire into the Questionnaire Star platform to generate an electronic questionnaire. Then, we distributed the electronic questionnaire to doctoral students who are currently studying through convenient sampling and snowball sampling methods with the help of social media such as WeChat. The data were collected from October 2022 to November 2022. A total of 605 doctoral students from 25 universities in mainland China participated in this survey voluntarily. After excluding invalid questionnaires with missing or contradictory information, 538 valid questionnaires were retained, resulting in an effective recovery rate of 88.9%.

Regarding the demographics of the valid sample, there were 246 (45.7%) male students and 292 (54.3%) female students. In terms of age, there were 189 (35.1%) doctoral students under 27 years old, 222 (41.3%) doctoral students between 28 and 33 years old, and 127 (23.8%) doctoral students over 34 years old. In terms of academic disciplines, 34.1% were from the humanities, 24.5% were from the social sciences, and 41.4% were from science, technology, agriculture, and medical science. In terms of university level, 349 students (64.9%) were from “double first-class” universities (refer to universities selected into first-class universities or first-class discipline construction universities in China), while 189 (35.1%) doctoral students were from “non-double first-class” universities. Regarding supervisor characteristics, male supervisors constituted 70.1%, while female supervisors made up the remaining 29.9%. In terms of the academic titles of supervisors, associate professors accounted for 19.4%, while professors comprised 80.6% of the sample.

### Measures

To ensure the content validity of the study, the concepts of challenge research stressors, achievement motivation, research creativity, and supervisor developmental feedback used in this study were derived from mature scales developed by scholars. The English version questionnaire was translated into Chinese through translation-back-translation. While avoiding distortion of the questionnaire, appropriate modifications were made to the wording and language order of the questionnaire to ensure that the questionnaire used in this survey conforms to Chinese linguistic habits. Prior to the formal survey, a pre-survey was conducted with relevant personnel from the target population. The questionnaire was adjusted and modified according to the opinions of the respondents, in order to improve the accuracy of the language used in the questionnaire and the standardization of its design, thereby further enhancing the content validity of the study. The details of the measurement items of the four variables are presented in Supplementary Appendix 1. All scale questions were measured in the form of a five-point Likert scale, ranging from 1 (strongly disagree) to 5 (strongly agree). Higher numbers indicated higher levels of agreement.

Research creativity was assessed using a scale adapted from Zhou and George (2001), with slight modifications to better suit the context of doctoral education. The final scale consisted of six items, including “I can propose original and practically significant research questions” and “I can interpret the research questions from a new perspective.” The Cronbach’s α coefficient of this scale in this study was 0.878.

Challenge research stressors were measured using a scale adapted from Cavanaugh et al. (2000), with slight adjustments made to account for specific challenge stressors experienced by doctoral students. The final scale consisted of five items, including “I have a large amount of research tasks to complete” and “I often feel the pressure of time in my research work.” The Cronbach’s α coefficient of this scale in this study was 0.877.

Achievement motivation was measured using a scale developed by Man et al. (1994), with minor modifications made to align it with the research context of doctoral students. The final scale consisted of six items, such as “I like novel and difficult research tasks and am willing to take risks” and “I will be attracted to research projects where the outcome of success is uncertain.” The Cronbach’s α coefficient of this scale in this study was 0.876.

Supervisor developmental feedback was assessed using a scale adapted from Zhou (2003), with slight modification to align with the characteristics of academic supervisors. The final scale consisted of three items, such as “My supervisor provides me with useful information on how to improve my research performance” and “While giving me feedback, my supervisor focuses on helping me to learn and improve.” The Cronbach’s α coefficient of this scale in this study was 0.766.

Control variables: Previous research has shown that personal characteristics of doctoral students, such as gender, age, disciplinary categories, university level, as well as the gender and academic title of their supervisors, may influence their research creativity. This study also included them as control variables.

### Data analysis

This study primarily utilized SPSS 26.0 and Mplus 7.4 software for data analysis. SPSS26.0 software is used for reliability and validity testing, descriptive statistics, correlation analysis, hierarchical regression analysis, and common method biases testing. The Mplus software is used for conducting confirmatory factor analysis (CFA), including estimating the standardized factor loadings of each item, testing convergent validity and discriminant validity of the four variables, and also examining the fit of the hypothetical model. Additionally, the bias-corrected non-parametric percentile bootstrap test method was used with the help of Mplus software to test the significance of the mediation effects and mediated moderation effects of the hypothetical model.

## Results

### Common method biases testing and confirmatory factor analysis

Considering that the data was obtained from teachers’ self-reports, there was a potential for common method bias. Therefore, this study utilized an anonymous questionnaire survey. An exploratory factor analysis was subsequently conducted on all scale items using Harman’s single-factor test. The results revealed that there were four factors with eigenvalues greater than one, and the first factor only explained 22.69% of the variance, which is significantly below the 40% threshold. Additionally, the results of the one-factor model confirmatory factor analysis presented in Table 1, indicated a poor model fit with *X*^2^/*DF* = 4.393, RMSEA = 0.123, IFI = 0.758, TLI = 0.727, CFI = 0.756, GFI = 0.697. These results suggest that common method bias was not a serious concern in the current study.

**Table 1:** Results of confirmatory factor analysis of hypothetical and competing models.

Models	*X* ^2^	*DF*	*X*^2^/*DF*	SRMR	RMSEA	IFI	CFI	TLI	GFI
four-factor model CRS, RC, AM, SDF	255.4	164	1.557	0.038	0.050	0.962	0.961	0.955	0.901
three-factor model CRS, RC + AM, SDF	349.1	167	2.090	0.044	0.069	0.924	0.923	0.912	0.850
two-factor model CRS + SDF + AM, RC	622.3	169	3.682	0.065	0.109	0.810	0.808	0.784	0.746
one-way model CRS + RC + AM+SDF	746.6	170	4.393	0.071	0.123	0.758	0.756	0.727	0.697

*N* = 538. CRS stands for Challenge Research Stressors, RC stands for Research Creativity, AM stands for Achievement Motivation, and SDF stands for Supervisor Developmental Feedback.

Additionally, as shown in Table 1, the fit index of the proposed four-factor model (*X*^2^/*DF* = 1.557; SRMR = 0.038; RMSEA = 0.050; IFI = 0.962; TLI = 0.955; CFI = 0.961; GFI = 0.901) significantly outperformed those of the other three alternative models, indicating that the hypothesized model is a better fit for the data in this study and the concepts of the four factors are mutually independent and have good discriminant validity.

### Reliability and validity testing

In this study, we assessed the reliability of the scale using SPSS 26.0 (Table 2). The Cronbach’s α coefficients of the four scales exceeded 0.7, indicating a high level of internal consistency. The KMO values, which exceeded 0.7 (*p* < 0.001), confirmed their suitability for factor analysis. Second, the standardized factor loadings of each item in the four variables exceeded 0.5, ranging from 0.533 to 0.825; the CR values of the four variables were all greater than 0.7; the AVE values were all greater than 0.5, demonstrating that the four scales have good convergent validity. In addition, the square root of AVE for each variable was higher than the Pearson correlation coefficient between variables (Table 3), which indicates a strong discriminant validity among the four constructs again.

**Table 2:** Results of reliability and validity test.

Variables	Factor loadings		*p*-value	CR	AVE	Cronbach’s α	KMO
Challenge Research Stressors	CRS1	0.697	<0.001	0.88	0.59	0.874	0.860
	CRS2	0.807	<0.001				
	CRS3	0.749	<0.001				
	CRS4	0.815	<0.001				
	CRS5	0.773	<0.001				
Research Creativity	RC1	0.770	<0.001	0.88	0.55	0.878	0.899
	RC2	0.757	<0.001				
	RC3	0.664	<0.001				
	RC4	0.694	<0.001				
	RC5	0.731	<0.001				
	RC6	0.817	<0.001				
Achievement Motivation	AM1	0.752	<0.001	0.88	0.55	0.876	0.868
	AM2	0.790	<0.001				
	AM3	0.801	<0.001				
	AM4	0.796	<0.001				
	AM5	0.731	<0.001				
	AM6	0.533	<0.001				
Supervisor Developmental Feedback	SDF1	0.825	<0.001	0.79	0.56	0.788	0.700
	SDF2	0.746	<0.001				
	SDF3	0.663	<0.001				

*N* = 538. CR: composite reliability; AVE: average variance extracted.

**Table 3:** Descriptive statistics and inter-correlations.

Variables	Means	SD	1	2	3	4
1. Challenge research stressors	3.77	0.75	**0.77**			
2. Research creativity	3.58	0.71	0.558***	**0.74**		
3. Achievement motivation	3.55	0.71	0.615***	0.720***	**0.74**	
4. Supervisor developmental feedback	4.21	0.68	0.373***	0.289*	0.329**	**0.75**

*N* = 538. *** indicates *p* < 0.001, ** indicates *p* < 0.01, * indicates *p* < 0.05 (two-tailed test). The bold number is the square root of AVE.

### Descriptive statistics and correlation analysis

Table 3 presents the means, standard deviations, and correlation coefficients of the variables. The results show that the mean value of challenge research stressors is 3.77, which is higher than the median value of 3. This signifies that doctoral students typically experience high research pressure. Additionally, the research creativity score has a mean value of 3.58, indicating a relatively high level of self-assessed creativity among doctoral students. The mean value of achievement motivation is 3.55, which is higher than the median value of 3. This suggests potential for improvement in students’ motivation levels. Additionally, the mean value of supervisors’ developmental feedback is 4.21, which is significantly higher than the median value of 3, indicating that doctoral students generally receive more extensive developmental feedback from their supervisors. In addition, there is a significant positive correlation between challenge research stressors, research creativity, achievement motivation, and supervisor developmental feedback. This correlation provides data support for the subsequent hypothesis testing.

### Hypothesis testing

#### Main effect and mediating effect testing

Before conducting hierarchical regression analysis, we performed a normality test on the data and found that the data is generally normally distributed (see Supplementary Appendix 1), making it suitable for regression analysis. The regression results are presented in Table 4. Challenge research stressors had a significant positive effect on research creativity (Model 5, β = 0.536, *p* < 0.001), supporting H1. Moreover, Challenge research stressors were found to have a significant positive effect on achievement motivation of doctoral students (Model 2, β = 0.592, *p* < 0.001). When achievement motivation was introduced as a mediator alongside challenge research stressors, the predictive effect of challenge research stressors on research creativity (Model 6, β = 0.188) decreased compared to Model 5 (β = 0.538), but it remained statistically significant. Additionally, achievement motivation significantly and positively predicted research creativity (Model 6, β = 0.586, *p* < 0.001), providing preliminary support for H2. To further evaluate the significance of the mediating effect, this study utilize the bias-corrected non-parametric percentile Bootstrap estimation method with 5,000 random samples to test the 95% confidence interval. If the confidence interval does not contain 0, the mediating effect is considered significant. As shown in Table 5, the direct effect of challenge research stressors on research creativity was 0.189, with a 95% confidence interval of [0.068, 0.288], excluding 0, indicating the significance of the direct effect. Meanwhile, achievement motivation exhibited a mediating effect of 0.347, with a 95% confidence interval of [0.261, 0.444], which does not include 0, indicating a significant mediating role of achievement motivation between challenge research stressors and doctoral students’ research creativity. The mediating effect accounts for 64.74% of the total effect (0.536), providing further statistical support for H2.

**Table 4:** Results of regression analysis.

Variables	Achievement Motivation				Research creativity				
	Model 1	Model 2	Model 3	Model 4	Model 5	Model 6	Model 7	Model 8	Model 9
Gender	−0.060	−0.065	−0.062	−0.052	−0.004	0.031	−0.009	0.000	0.029
Age	0.232**	0.133*	0.139*	0.141*	0.113	0.022	0.105	0.107	0.027
Discipline	0.075	0.045	0.049	0.044	0.060	0.027	0.056	0.052	0.027
Institution level	−0.057	−0.066	−0.089	−0.090	−0.056	−0.013	−0.072	−0.073	−0.022
Gender of supervisor	−0.073	−0.107	−0.107*	−0.095	−0.040	0.022	−0.047	−0.036	0.017
Academic title of supervisor	−0.059	−0.070	−0.073	−0.092	−0.047	−0.006	−0.054	−0.072	−0.019
Challenge research stressors		0.592***	0.541***	0.536***	0.536***	0.188**	0.494***	0.489***	0.185**
Supervisor developmental feedback			0.136*	0.184**			0.112	0.156*	0.052
Achievement Motivation						0.586***			0.567***
Challenge research stressors* supervisor developmental feedback				0.151**				0.140*	0.055
*R* ^2^	0.100	0.437	0.452	0.472	0.352	0.546	0.363	0.380	0.549
Adjusted *R*^2^	0.071	0.416	0.430	0.448	0.329	0.527	0.336	0.351	0.526
△*R*^2^	0.100	0.337	0.015	0.020	0.276	0.194	0.286	0.017	0.169
*F*	3.467**	21.144***	19.908***	19.321***	14.827***	28.995***	13.726***	13.238***	23.831***

*N* = 538.

**Table 5:** Bootstrap test for mediating effects.

	Model paths	Estimated effect	Standard error	95% Confidence intervals	Effect size
Challenge research stressors → Achievement motivation → Research creativity	Total effect	0.536	0.053	[0.402, 0.609]	
	direct effect	0.189	0.056	[0.068, 0.288]	35.26%
	Indirect effect	0.347	0.047	[0.261, 0.444]	64.74%

*N* = 538.

#### Moderating effect of supervisor developmental feedback testing

To avoid multicollinearity issues, we mean-centered the variables of challenge research stressors and supervisor developmental feedback, and then created their interaction term. We sequentially included control variables, independent variable, moderator variable, and interaction term in the multilevel linear regression analysis with achievement motivation and research creativity as dependent variables, respectively. The results in Table 4 demonstrate that the interaction terms of challenge research stressors and supervisors’ developmental feedback had a significant and positive effect on the doctoral students’ achievement motivation (Model 4, β = 0.151, *p* < 0.01) and research creativity (Model 8, β = 0.140, *p* < 0.05), supporting H3 and H4.

**Figure 2:**
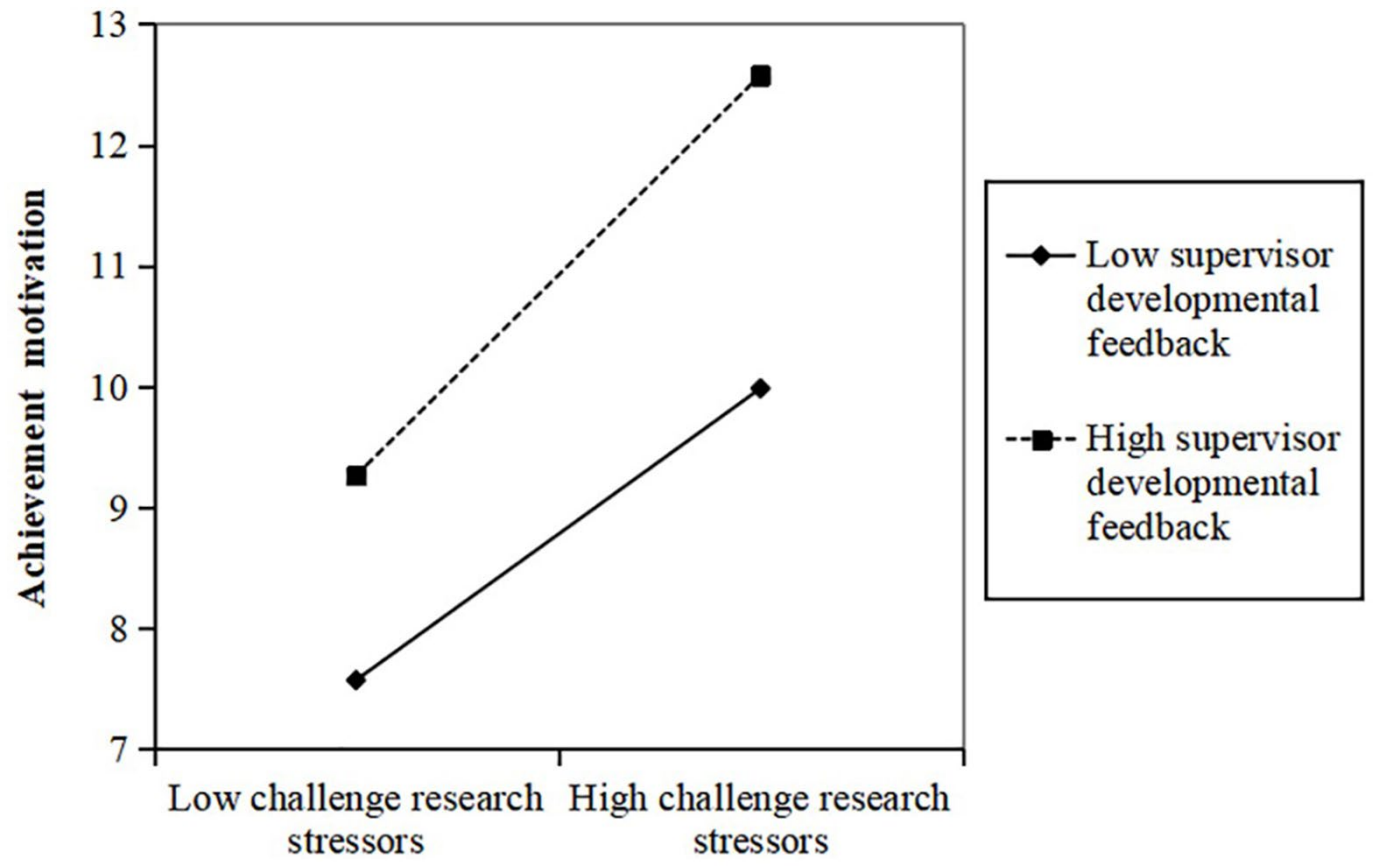
Moderating effect of supervisor developmental feedback between challenge research stressors and achievement motivation.

**Figure 3:**
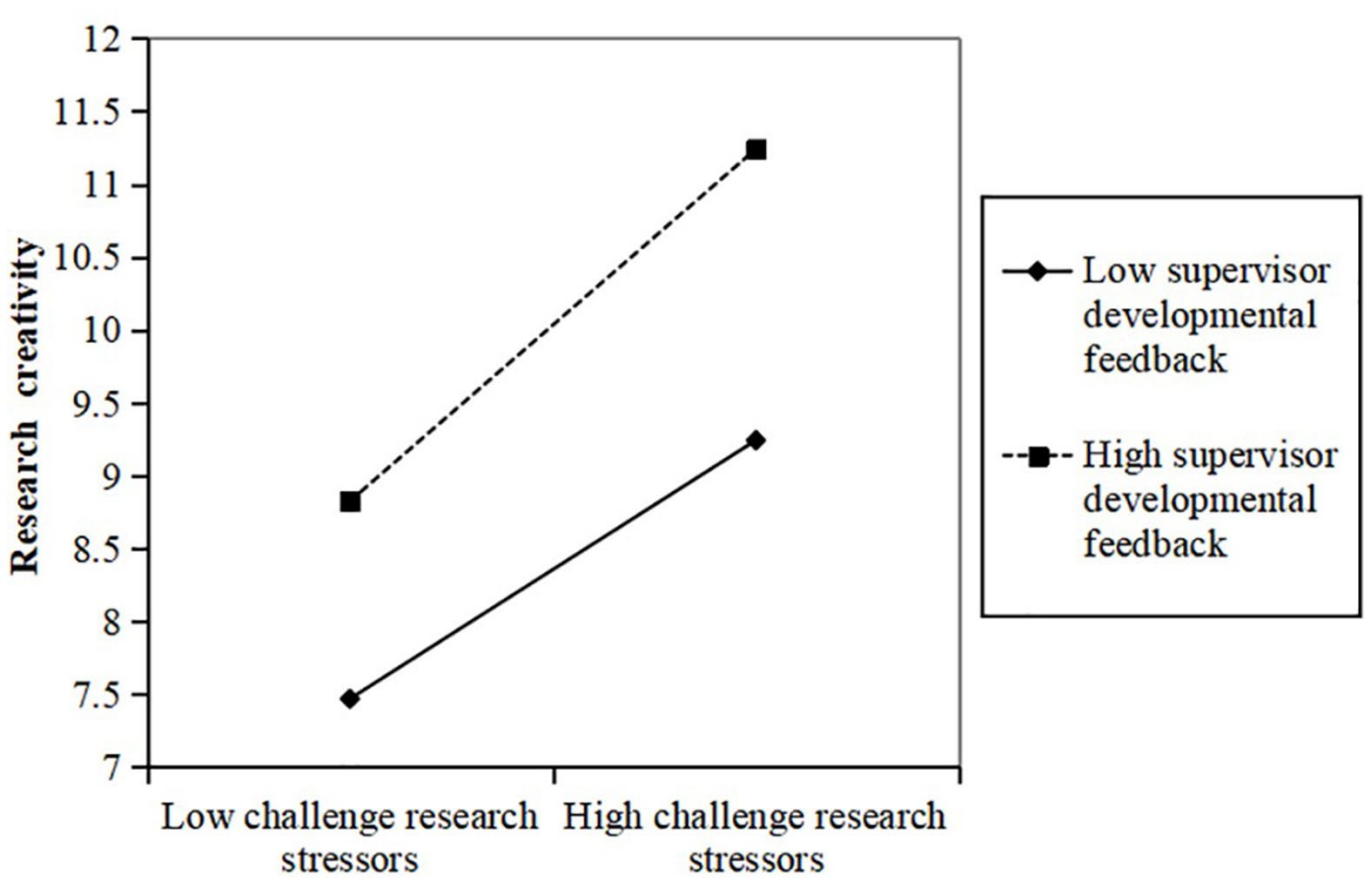
Moderating effect of supervisor developmental feedback between challenge research stressors and research creativity.

To examine the moderating impact of different levels of supervisor developmental feedback on the relationship between challenge research stressors and the achievement motivation and research creativity of doctoral students, a simple slope test was conducted and graphed. Supervisor developmental feedback was dichotomized into high and low groups based on one standard deviation above and below the mean, following the approach recommended by Preacher et al. (2006). As showed in Figure 2, the positive impact of challenge research stressors on the achievement motivation of doctoral students was significantly greater in the presence of high supervisor developmental feedback (β = 0.643, *p* < 0.001) compared to low supervisor developmental feedback (β = 0.369, *p* < 0.001). This finding suggests that supervisor developmental feedback effectively moderates the relationship between challenge research stressors and doctoral students’ achievement motivation, providing further support for H3. Similarly, as shown in Figure 3, the positive effect of challenge research stressors on doctoral students’ research creativity was significantly more pronounced in the context of high supervisor developmental feedback (β = 0.589, *p* < 0.001) compared to low supervisor developmental feedback (β = 0.334, *p* < 0.001). This outcome indicates that supervisors’ developmental feedback positively moderates the relationship between challenge research stressors and doctoral students’ research creativity, further supporting H4.

#### Mediated moderation effect of supervisor developmental feedback testing

The present study applies the sequential test of the mediated moderation effect proposed by Edwards and Lambert (2007). Firstly, we assessed the significance of the regression coefficient of the interaction term (challenge research stressors * supervisor developmental feedback) in relation to research creativity. Then we tested whether the coefficient of the interaction term on the mediator (achievement motivation) was significant. The above two steps have been supported by H3 and H4. Finally, we tested whether the coefficient for the mediator variable (achievement motivation) remained significant when added to the model exploring the moderating effect of supervisor developmental feedback on the relationship between challenge research stressors and research creativity. As shown in model 9 of Table 4, we observed that a significant and positive effect of achievement motivation on doctoral students’ research creativity (β = 0.567, *p* < 0.001). However, the interaction term (challenge research stressors*supervisor developmental feedback) no longer showed a significant effect on research creativity (β = 0.05, *p* > 0.055). These findings suggest that the moderating effect of supervisor developmental feedback on the relationship between challenge research stressors and doctoral students’ research creativity was fully mediated by achievement motivation. Therefore, H5 was supported.

To further examine the significance of achievement motivation as a mediator in the moderating effect of supervisors’ developmental feedback on the relationship between challenge research stressors and doctoral students’ research creativity, this study utilized the Bootstrap method. The sample was randomly replicated 5,000 times using Mplus7.4 software to analyze the overall model. We evaluated the 95% confidence intervals for the mediated moderation effect, deeming it significant if these intervals did not include 0. The results of this analysis showed that the indirect effect of the interaction of challenge research stressors and supervisor developmental feedback on doctoral students’ research creativity through achievement motivation was 0.297 (*p* < 0.001). The 95% confidence interval was [0.217, 0.401], which did not include 0, suggesting that achievement motivation mediates the interaction effect of challenge research stressors and supervisor developmental feedback on doctoral students’ research creativity, providing additional support for H5. As indicated in Table 6, the mediating effect of achievement motivation on the relationship between challenging research pressure and doctoral students’ research creativity was found to be significant at both high levels of supervisor developmental feedback (effect value = 0.377, 95% confidence interval [0.268, 0.510]) and low levels of supervisor developmental feedback (effect value = 0.216, 95% confidence interval [0.117, 0.342]). Furthermore, the difference between high and low levels was significant, with a 95% confidence interval of [0.024, 0.303]. This result confirms the validity of the mediated moderation model proposed in this study.

**Table 6:** Bootstrap test for mediated moderation effects.

	Supervisor developmental feedback	Estimated effect	Standard error	95% Confidence intervals
Challenge research stressors → Achievement motivation → Research creativity	*M*–1SD	0.216	0.058	[0.117, 0.342]
	*M* + 1SD	0.377	0.061	[0.268, 0.510]
	The difference	0.161	0.072	[0.024, 0.303]

*N* = 538.

## Discussion

Based on the Job Demands-Resources Model and achievement motivation theory, this study explores the relationship between challenge research stressors and doctoral students’ research creativity, as well as the underlying mechanism. Using a sample of 538 Chinese doctoral students, we found that challenge research stressors, supervisor developmental feedback, achievement motivation, and research creativity form a mediated moderation model. Challenge research stressors have a direct impact on the research creativity of doctoral students, and this influence is moderated by supervisor developmental feedback and partially mediated by achievement motivation. Moreover, doctoral students’ achievement motivation fully mediates the interaction effect of supervisor developmental feedback and challenge research stressors on the research creativity of doctoral students.

First, the present study found that challenge research stressors significantly and positively predicted doctoral students’ research creativity, which was consistent with the prior research (Ohly and Fritz, 2010; Wang et al., 2014; Gu and Chang, 2021). The Expectancy-Value Theory highlights how an individual’s motivation for completing a task depends on their perception of the likelihood of success and the value of task. In other words, the higher an individual perceives the likelihood of achieving a goal, the greater the incentive value derived from it, and the stronger their motivation becomes to accomplish the task (Wigfield and Eccles, 2000). When doctoral students encounter stressors associated with challenging research demands, they carefully assess the effort required to cope with these stressors and the potential rewards (Lazarus and Folkman, 1986). They are likely to respond positively to challenge research stressors because these stressors are manageable and achievable research tasks. This allows them to successfully cope with stressors through their own efforts (Crawford et al., 2010). Overcoming such stressors will lead to beneficial research outcomes, such as academic growth or improved research capabilities (Lepine et al., 2004). Therefore, doctoral students’ sense of competence and control increases during the process of dealing with challenge research stressors. This, in turn, enhances their intrinsic motivation and dedication to academic research, ultimately stimulating them to propose innovative topics and address research problems creatively. Furthermore, based on the categorization of stressors as “good” or “bad” by Cavanaugh et al. (2000) and Rodell and Judge (2009), challenge stressors are classified as the “good.” Doctoral students’ positive perception of challenge research stressors elicits positive emotions and enhances their research self-efficacy (Travis et al., 2020), which, in turn, promotes cognitive flexibility and encourages the pursuit of novel problem-solving approaches, ultimately enhancing their research creativity.

Second, the results of the present study showed that doctoral students’ achievement motivation partially mediated the relationship between challenge research stressors and research creativity. This result is in line with the previous study highlighting intrinsic motivation’s key role in mediating the impact of external environmental factors on individuals’ creativity (Svensson, 2015; Gao et al., 2020). Meanwhile, the finding that challenge research stressors positively predicted doctoral students’ achievement motivation was consistent with the previous study (Yao and Ma, 2021), and also validated the motivational activation characteristics of challenge stressors proposed by Lepine et al. (2004). It is generally believed that challenge research stressors can be overcome through hard work and can ultimately lead to positive results (LePine, 2022). Therefore, challenge stressors are likely to stimulate the intrinsic motivation of doctoral students to achieve their research goals. Additionally, our finding that achievement motivation significantly predicted doctoral students’ research creativity was also consistent with the conclusion of existing research (Schoen, 2015). Doctoral students with high achievement motivation tend to enjoy more challenging tasks, set higher goals, and be more persistent in the face of risky and uncertain creative activities (Dweck, 1986). Therefore, they are more likely to prioritize learning new knowledge and skills related to the tasks, and employ divergent thinking to propose solutions to problems (Gao et al., 2020).

Third, this study found that supervisor developmental feedback, as a supportive contextual resource, positively moderated the impact of challenge research stressors on doctoral students’ achievement motivation. That is, the more developmental feedback received from supervisors, the greater positive impact of challenge research stressors on students’ achievement motivation. This result is consistent with previous research findings (Vansteenkiste et al., 2009; Dupont et al., 2015), which indicate that accurate feedback and advice from supervisors can enhance students’ perceived competence and academic engagement, as well as intrinsic motivation. At the same time, this result validated the fundamental hypothesis of the job demands-resources model: in high-demand situations, sufficient job resources, such as supervisor feedback, can mitigate the negative impact of job demands and enhance individuals’ work engagement and motivation levels (Demerouti and Bakker, 2011). Specifically, when challenge research stressors become routines that doctoral students must confront, supervisor developmental feedback not only imparts valuable research insights but also signifies encouragement and support (Shang et al., 2023). This instrumental support and emotional support bolster doctoral students’ sense of meaning, efficacy, and belonging in research, and also serve as a counterbalance to the depletion of cognitive and emotional resources triggered by challenging demands (Bakker et al., 2007), which helps students effectively manage challenge research stressors and consequently enhance their intrinsic research motivation and academic engagement (Crawford et al., 2010).

Meanwhile, this study also found that supervisor developmental feedback moderated the impact of challenge research stressors on doctoral students’ research creativity. In other words, the more developmental feedback doctoral students received from their supervisors, the greater positive influence of challenge research stressors on their research creativity. On one hand, supervisor developmental feedback not only provides doctoral students with valuable research resources that equipped them to cope with challenging research demands, but also motivates doctoral students to put forward novel ideas and address complex research tasks creatively (Shang et al., 2023). On the other hand, supervisor developmental feedback, as non-performance-oriented informational feedback, helps to alleviate students’ perceived stress and foster a relaxed research atmosphere (Li et al., 2011). This environement encourages doctoral students to engage in research out of their genuine interest in science (Joo and Park, 2010). As a result, it enhances doctoral students’ divergent thinking and improves their research creativity (Runco and Acar, 2012).

Fourth, this study further found that achievement motivation fully mediated the interaction effect of supervisor developmental feedback and challenge research stressors on doctoral students’ research creativity. This finding further validated the hypothesis of the JD-R model theory: the interaction effect of high job demands and high job resources has the most significant motivational effect on individuals (Bakker et al., 2007). Under high challenging research demands, doctoral students who had access to abundant research resources were more proactive in their engagement in research work and exhibited higher level of academic enthusiasm, which triggered a “motivation activation process” (Bakker and Demerouti, 2014). In other words, doctoral students facing challenge research stressors are eager to obtain more resources, the valuable information resources provided by their supervisors’ developmental feedback can better stimulate their intrinsic motivation to pursue academic achievements, thus promoting their engagement in research activities creatively. Therefore, the positive moderating effect of supervisor developmental feedback on the relationship between challenge research stressors and doctoral students’ research creativity was completely mediated by achievement motivation.

### Theoretical contributions

This study has certain theoretical contributions. Firstly, it clarifies the relationship between challenge research stressors and research creativity from a new theoretical perspective. Previous research has predominantly examined the relationship between challenge stressors and individual creativity from the perspectives of organizational support, emotion, job involvement, and cognitive evaluation, often overlooking the role of individual intrinsic motivation. Based on achievement motivation theory, this study introduces the mediating effect of achievement motivation. It reveals that achievement motivation is a crucial psychological mechanism for explaining the influence of challenge research stressors on the research creativity of doctoral students. Therefore, this finding deepens our understanding of the specific pathway through which challenge stressors affect individual creativity. Moreover, from the perspective of achievement motivation, this study considers challenge research stressors as a beneficial aspect of the research environment and confirms their positive influence on the research creativity of doctoral students, specifically in the Chinese context. This extension of stressors research effectively prompts the academic community to reconsider the impact of challenge research stressors.

Secondly, this study contributes to the existing research on the influence of supervisor behavior on the research creativity of doctoral students. Previous research has primarily focused on various supervisory guidance styles, such as paternalistic (Su et al., 2021), inclusive (Zhang et al., 2023), and abusive styles (Cohen and Baruch, 2022). However, these studies have overlooked the significance of supervisor developmental feedback in stimulating doctoral students’ research creativity, particularly in the context of challenge research stressors. In today’s context where diverse supervisory styles coexist, it is essential to recognize the distinctive role of supervisor developmental feedback compared to the above supervisory styles, since feedback implies communication and interaction between doctoral students and supervisors, which will facilitate the emergence of innovative ideas, thus enhancing the creativity of doctoral students.

Thirdly, this study provides additional empirical evidence to support and validate the underlying principles of the JD-R Model, which confirms that the development and maintenance of doctoral students’ research creativity are influenced by the interaction of environmental, supervisor, and individual factors. Moreover, this study expands the scope of the JD-R Model by demonstrating its relevance and applicability in the context of doctoral education.

### Practice implications

Based on the above conclusions and discussions, this study presents the following practical recommendations. First, our findings show that challenge research stressors are positively related to doctoral students’ achievement motivation and research creativity. Consequently, supervisors should properly assign diverse and challenging tasks to doctoral students based on their research abilities and interests. They should also set reasonable and challenging research demands that provide rewarding research experiences. This can be achieved by controlling the completion time of research tasks, increasing the workload of research, and raising the innovation requirements of research. However, when setting research demands, supervisors should provide timely research support to create a “high demands-high support” motivating and support mechanism, which is important in order to avoid causing anxiety to doctoral students due to excessively challenging research requirements. Moreover, supervisors should acknowledge the individual variations in stress management abilities of doctoral students. They should pay attention to the emotional fluctuations and behavioral responses of these students, and particularly focus on satisfying their basic psychological needs for research efficacy, belonging, and autonomy. By doing so, supervisors can stimulate the academic motivation and research creativity of doctoral students. Furthermore, since encountering challenge research stressors is inevitable for doctoral students in their research work, it is crucial to promote a proper understanding of these stressors and enhance students’ psychological resilience. Academic institutions should provide regular psychological counseling and psycho-educational courses to help doctoral students learn how to actively cope with challenge research stressors.

Second, since achievement motivation positively predicts the research creativity of doctoral students, supervisors and departments can take steps to enhance it. Previous studies have shown that a supportive academic atmosphere and learning environment are key elements in enhancing students’ achievement motivation (Baeten et al., 2013; Huang et al., 2022). Therefore, it is important to create a supportive and tolerant academic environment in which doctoral students are encouraged to address research challenges. For example, creating an inclusive climate for choosing research topics, establishing novel evaluation methods that are appropriate for innovative research, recognizing and supporting individuals who are willing to confront challenges in scientific research, even in the face of setbacks. Moreover, providing various forms of research support, including academic, interpersonal, and emotional support, can help doctoral students alleviate the stress associated with their research. Excessive research pressure can erode feelings of competence and diminish motivation. However, positive social support can fulfill individuals’ basic psychological needs for competence, autonomy, and belonging, and then stimulate their motivation to achieve research goals (Chen et al., 2019). Therefore, establishing a learning support system consisting of teachers, counselors, senior teaching assistants, and classmates would be very helpful in alleviating stress among doctoral students and promoting their achievement motivation. In addition, helping doctoral students gain a sense of achievement by providing them with opportunities for success. For example, providing additional encouragement, recognition, and guidance in their presentation and implementation of creative ideas can empower doctoral students to actively engage in creative activities and enhance their research creativity through practical experience.

Third, supervisor developmental feedback is a crucial external resource for maintaining the achievement motivation of doctoral students and fostering their research creativity in a highly competitive and challenging research environment. Therefore, supervisors should provide timely academic guidance and professional feedback, offering valuable insights to enhance doctoral students’ research abilities, which will bolster their self-confidence and reinforce their drive for success. In addition, supervisors should provide timely emotional feedback, such as emotional care and psychological support, to help alleviate the confusion and uncertainty caused by academic pressure in doctoral students. This will also help strengthen their psychological resilience and foster a sense of belonging within a team. Consequently, their intrinsic academic motivation is strengthened, ultimately driving their enthusiastic involvement in research endeavors. Furthermore, academic institutions should prioritize enhancing emotional intelligence and interpersonal skills training for supervisors, and also optimize and improve the feedback methods used by supervisors. This will effectively help doctoral students cope with academic pressure, stay committed to their academic aspirations, and maintain their passion for academia.

### Limitations and future direction

Although this study contributes to the existing research on the relationship between research stressors and research creativity of doctoral students, there are also some limitations. Firstly, it uses a cross-sectional design and data, which limits its ability to establish causality. Future research could employ longitudinal methods to further explore the topic. Secondly, this study analyses challenge stressors from an integration paradigm perspective. In other words, this study examines the various dimensions of challenge stressors as a whole. However, some scholars in the academic community argue that the dimensions of challenge stressors, such as time pressure and workload, should be discussed separately (Qin et al., 2022). Therefore, future research can explore the mechanisms and boundary conditions of different types of challenge stressors on individual creativity. Lastly, since all variables in this study were assessed by the doctoral students themselves, there’s a potential for social desirability bias and common method bias. Future studies could incorporate more objective measures, such as supervisor evaluations, to improve rigor and credibility.

## Conclusion

The current study shows that challenge research stressors faced by doctoral students have a positive influence on their research creativity. Particularly, this influence is moderated by supervisor developmental feedback. Moreover, our results suggest a mediated moderation model, in that, achievement motivation not only partially mediates the influence of challenge research stressors on the research creativity of doctoral students, but also fully mediates the interaction effect of supervisor developmental feedback and challenge research stressors on doctoral students’ research creativity.

## Data availability statement

The original contributions presented in the study are included in the article/Supplementary material, further inquiries can be directed to the corresponding author.

## Ethics statement

The studies involving humans were approved by Ethical Approval of Hunan Normal University Biomedical Research Ethics Committee. The studies were conducted in accordance with the local legislation and institutional requirements. The participants provided their written informed consent to participate in this study.

## Author contributions

CL: Writing – original draft, Conceptualization, Data curation, Formal analysis, Methodology. MW: Writing – review & editing, Investigation, Software, Supervision. XG: Project administration, Supervision, Writing – review & editing.
