# Supplementary material for: The influence of challenge research stressors on research creativity among Chinese doctoral students: a mediated moderation model
Source: Front Psychol. 2023 Nov 1;14:1290342. doi: 10.3389/fpsyg.2023.1290342 (PMC10646261; doi:10.3389/fpsyg.2023.1290342)
Supplement: Supplementary file 1 [file Table_1.DOCX]

**Supplementary Appendix 1.** Detailed questionnaire items and Normality test

| **Variables** | **Items** | **Means** | **Skewness** | **Kurtosis** |
| --- | --- | --- | --- | --- |
| Research Creativity | I can propose original and practically significant research questions. | 3.72 | -0.083 | -0.787 |
|  | I can interpret the research questions from a new perspective. | 3.62 | -0.083 | -0.502 |
|  | I can propose new pathways, methods, processes, or means to break through the research dilemma. | 3.50 | -0.140 | -0.702 |
|  | I can skillfully utilize research techniques, research tools, or experimental equipment. | 3.52 | -0.141 | -0.289 |
|  | I can discover new evidence to answer the research questions in the study. | 3.58 | -0.178 | -0.280 |
|  | I can summarize the research findings and propose new theories that can be generalized. | 3.52 | 0.025 | -0.845 |
| Challenge Research Stressors | The academic requirements for graduation in my field are very high. | 3.98 | -0.528 | 0.004 |
|  | I have a large amount of research tasks to complete. | 3.71 | -0.260 | -0.382 |
|  | I often feel the pressure of time in my research work. | 3.90 | -0.546 | -0.189 |
|  | In the research group, I need to take on important responsibilities. | 3.49 | -0.364 | -0.260 |
|  | Engaging in scientific research requires me to master various research methods. | 3.74 | -0.355 | -0.068 |
| Achievement Motivation | I enjoy persistently studying challenging research questions. | 3.47 | -0.063 | -0.598 |
|  | I like novel and difficult research tasks and am willing to take risks. | 3.40 | -0.027 | -0.596 |
|  | I will be attracted to challenging tasks. | 3.22 | 0.023 | -0.543 |
|  | I am deeply attracted to research opportunities that can measure my own abilities. | 3.57 | -0.253 | -0.251 |
|  | I will be attracted to research projects where the outcome of success is uncertain. | 3.37 | 0.010 | -0.669 |
|  | I feel happy after successfully completing challenging research tasks through hard work. | 4.25 | -0.812 | 0.144 |
| Supervisor Developmental Feedback | My supervisor provides me with useful information on how to improve my research performance. | 4.23 | -0.812 | -0.604 |
|  | While giving me feedback, my supervisor focuses on helping me to learn and improve. | 4.23 | -1.018 | 1.047 |
|  | My academic supervisor often gives me developmental feedback. | 4.18 | -0.752 | 0.006 |
